# Falling down a flight of stairs: The impact of age and intoxication on injury pattern and severity

**DOI:** 10.1177/1460408617720948

**Published:** 2017-08-01

**Authors:** Hridesh Chatha, Ian Sammy, Michael Hickey, Abdo Sattout, John Hollingsworth

**Affiliations:** 1 Barnsley District General Hospital, Barnsley, UK; 2 Aintree University Hospital NHS Foundation Trust, Liverpool, UK; 3 School of Health and Related Research, The University of Sheffield, Sheffield, UK

**Keywords:** Multiple trauma, wounds and injuries, accidental falls, aged, emergency department, emergency services, hospital

## Abstract

**Background:**

Falling down a flight of stairs is a common injury mechanism in major trauma patients, but little research has been undertaken into the impact of age and alcohol intoxication on the injury patterns of these patients. The aim of this study was to compare the impact of age and alcohol intoxication on injury pattern and severity in patients who fell down a flight of stairs.

**Methods:**

This was a retrospective observational study of prospectively collected trauma registry data from a major trauma centre in the United Kingdom comparing older and younger adult patients admitted to the Emergency Department following a fall down a flight of stairs between July 2012 and March 2015.

**Results:**

Older patients were more likely to suffer injuries to all body regions and sustained more severe injuries to the spine; they were also more likely to suffer polytrauma (23.6% versus 10.6%; p < 0.001). Intoxicated patients were more likely to suffer injuries to the head and neck (42.9% versus 30.5%; p = 0.006) and were significantly younger than sober patients (53 versus 69 years; p < 0.001).

**Conclusion:**

Older patients who fall down a flight of stairs are significantly different from their younger counterparts, with a different injury pattern and a greater likelihood of polytrauma. In addition, alcohol intoxication also affects injury pattern in people who have fallen down a flight of stairs, increasing the risk of traumatic brain injury. Both age and intoxication should be considered when managing these patients.

## Introduction

While falling down a flight of stairs is increasingly recognised as a significant mechanism of injury in all age groups, there is little research on the impact of age on the associated epidemiology, injury pattern and injury severity. Patients that fall down a flight of stairs are often a challenge to manage for the trauma team. The fall is often unwitnessed, therefore the exact detail of the mechanism, and in particular the height fallen, is not available. Patients most at risk from this mechanism are either older patients or younger people who are intoxicated, each scenario providing unique diagnostic and management challenges.^1–5^ Older patients are more likely to have significant injuries despite a low risk mechanism, while the presence of comorbidities increases their risk of mortality despite a lower injury severity.^6^ Intoxicated patients also provide a diagnostic challenge, as physical examination is often unreliable.^7^

Previous authors have acknowledged the propensity for falls down a flight of stairs in older people and its significance for injury pattern. Boyé et al.^1^ investigating falls in older people in the Netherlands found that 11% of their sample fell while walking up or down stairs. However, this mechanism accounted for 51% of patients who sustained traumatic brain injuries, emphasising the clinical importance of this injury mechanism in older people.

Falls down a flight of stairs are known to cause serious injuries. Van Hensbroek et al.’s^2^ study of 464 patients who fell down a flight of stairs revealed a significant incidence of traumatic brain injury (5.6%), limb fractures (22.4%) and thoracic injuries (7.4%), while 13% of patients required admission, and seven were admitted to the intensive care unit.

Increasing age has previously been noted as a risk factor for falling down a flight of stairs in adults, but its effect on injury pattern and severity is not fully understood.^1,5^ This single-centre observational study of data from a major trauma centre in the United Kingdom aimed to determine the impact of age and alcohol intoxication on the pattern and severity of injuries sustained after falling down a flight of stairs.

## Methods

This retrospective observational study of prospectively collected trauma registry data included all adult patients who presented after falling down a flight of stairs between July 2012 and March 2015. Data were obtained from the Aintree Trauma Database, which consists of prospectively collected data from all patients presenting as trauma team activations to the Aintree University Hospital (AUH) emergency department. The hospital operates a single-level trauma team activation protocol; patients with a high energy injury mechanism (road traffic collisions, falls from a height, assault), significant penetrating injury (stabs and gunshots to the torso), obvious physical signs of serious or multiple injuries or abnormal vital signs (including Glasgow coma score, systolic blood pressure, oxygen saturations or respiratory rate) trigger a full trauma team activation.

AUH is the regional trauma centre in Merseyside, serving a population of approximately 1.4 million people. All major trauma patients from the surrounding Merseyside region are transferred to AUH for resuscitation and definitive management. There are approximately 700 trauma team activations per year; during the study period, the hospital received 2205 major trauma patients that required trauma team activation. Ethical approval was gained from the Health Research Authority of England (HRA) via the Integrated Research Application System (IRAS) for use of the AUH Trauma Database in anonymised research in March 2014 (IRAS project ID 140559).

All adult patients aged 16 and older entered onto the AUH trauma registry, who presented after falling down a flight of stairs, were included in this study. Patients with missing data were excluded from the analysis. Patients aged ≥65 years were compared to younger patients in relation to injury pattern and severity, polytrauma and alcohol intoxication. Injury severity was estimated for different body regions using the Abbreviated Injury Score (AIS) of the most severe injury for the designated regions,^8^ although some regions were combined for descriptive purposes in the study and then used to calculate Injury Severity Score (ISS).^9^ Polytrauma was defined as injuries of AIS ≥3 in two or more different body regions. Intoxicated patients were also compared to sober patients. Intoxication was determined on clinical assessment and history.

Descriptive statistics were used to present the demographic and clinical characteristics of both groups. Non-parametrically distributed data were described using medians and interquartile ranges, while parametrically distributed data were described using means and standard deviations. Categorical variables were compared using Chi-squared tests while continuous variables were compared using the Mann–Whitney U-test for non-parametric data and the Student’s t-test for parametric data. Analysis was performed in SPSS version 22 and Excel 2016.

## Results

**Figure 1.**
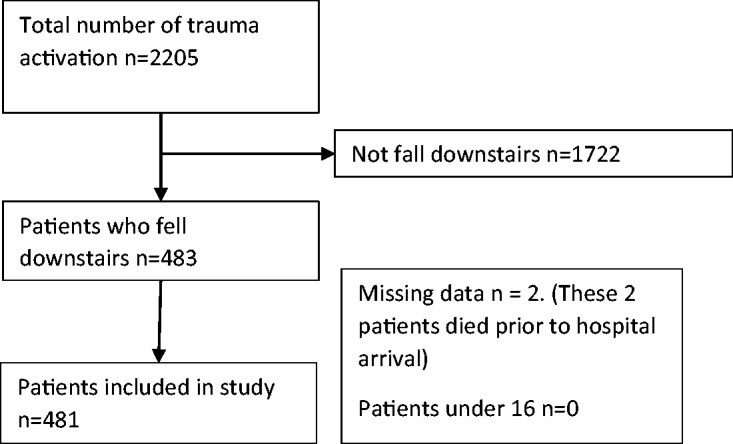
Recruitment flow diagram for the study.

During the study period 483 patients presented after falling down a flight of stairs, two were excluded as they suffered pre-hospital cardiac arrest and did not have any investigations in hospital leaving 481 patients in the final analysis (Figure 1). A summary of the patients’ demographic and clinical characteristics is shown in Table 1.

**Table 1.** Demographic and clinical characteristics of study participants.

	All data (n = 481) *n(% [95%CI])*	Age < 65 (n = 273) *n(% [95%CI])*	Age ≥ 65 (n = 208) *n(% [95%CI])*	p value
Age in years (median, IQR)	61.0 (IQR 46.0–75.0)	49.0 (36.0–58.5)	78.0 (70.0–84.0)	–
**Gender**				
Male	256 (53.1% [48.7–57.6%])	152 (55.7% [49.8–61.6%])	104 (50.0% [43.2–56.8%])	p = 0.220
Female	225 (46.9% [42.4–51.3%])	121 (44.3% [38.4–50.2%)	104 (50.0% [43.2–56.8%])	
**Alcohol intoxication**	196 (40.7% [36.6–45.0%])	154 (56.4% [50.5–62.3%])	42 (20.2% [14.7–25.6%])	p < 0.001
**Injury pattern (number of patients with injuries to each body region)**				
Head and face	171 (35.5% [31.2–39.7%])	82 (30.0% [24.6–35.5%])	89 (42.8% [36.1–49.5%])	p = 0.004
Chest	120 (24.9% [21.0–28.8%])	47 (17.2% [12.7–21.7%])	73 (35.1% [28.6–41.6%])	p < 0.001
Abdomen and pelvis	28 (5.8% [3.7–7.9%])	9 (3.3% [1.2–5.4%])	19 (9.1% [5.2–13.0%])	p = 0.007
Spine	107 (22.2% [18.5–25.9%])	39 (14.3% [10.1–18.4%])	68 (32.5% [26.3–39.1%])	p < 0.001
Limb	96 (19.9% [16.4–23.5%])	44 (16.1% [11.8–20.5%])	52 (25.0% [19.1–30.9%])	p = 0.016
Polytrauma	78 (16.2% [12.9–19.5%])	29 (10.6% [7.0–14.3%])	49 (23.6% [17.8–29.3%])	p < 0.001
**Injury severity (AIS; median and IQR)**				
Head and face	4 (IQR 2–5)	4 (IQR 3–5)	4 (IQR 2-5)	p = 0.080
Chest	3 (IQR 2-4)	3 (IQR 2–4)	3 (IQR 3–4)	p = 0.880
Abdomen and pelvis	2 (IQR 2–2)	3 (IQR 2–3)	2 (IQR 2–2)	p = 0.290
Spinal	2 (IQR 2–3)	2 (IQR 2- 2)	2 (IQR 2–3)	p < 0.001
Limb	2 (IQR 2–2)	2 (IQR 2-2)	2 (IQR 2–2)	p = 0.290
**Injury Severity Score**	14 (IQR 8–25)	13 (IQR 5- 25)	16 (IQR 9–25]	p = 0.130

AIS: Abbreviated Injury Score; CI: confidence interval; IQR: interquartile range.

The majority of patients were male. Compared to younger patients, older patients were more likely to sustain injuries in all body regions, particularly the head and face, chest and spine. Older patients also sustained more severe injuries to the spine and were more likely to sustain polytrauma than their younger counterparts (Table 1). Older people also had higher ISSs, but this was not statistically significant (median ISS 16 versus 13; p = 0.130).

**Figure 2.**
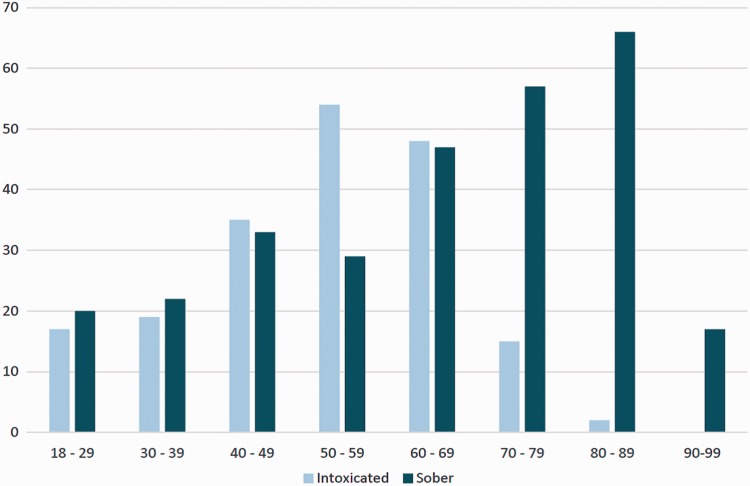
Age distribution of sober and intoxicated older patients, demonstrating that sober patients are significantly older than intoxicated patients.

Intoxicated patients were more likely to be male, and their median age was younger (Table 2 and Figure 2); 43% of intoxicated patients sustained head and neck injuries, compared to 30.5% of sober patients (p = 0.006), but injuries to the chest, abdomen and pelvis were less common in intoxicated patients, compared to sober patients (Table 2). There was no significant difference in ISS between intoxicated and sober patients.

**Table 2.** Comparison of demographic and clinical characteristic of sober and intoxicated patients.

	Intoxicated (n = 196) *n(% [95%CI])*	Sober (n = 285) *n(% [95%CI])*	p value
Age (median, IQR)	53 (IQR 43–63)	69 (IQR 50–81)	<0.001
**Gender**			
Male	128 (65.3% [58.6–72.0%])	128 (44.9% [39.1–50.7%])	<0.001
Female	68 (34.7% [28.0–41.4%])	157 (55.1% [49.3–60.9%])	
**Injury pattern (number of patients with injuries to each body region)**			
Head and face	84 (42.9% [35.9–49.8%])	87 (30.5% [25.2–35.9%])	0.006
Chest	40 (20.4% [14.8–26.1%])	80 (28.1% [22.9–33.3%])	0.056
Abdomen and pelvis	6 (3.1% [0.6–5.5%])	22 (7.7% [4.6–10.8%])	0.032
Spine	38 (19.4% [13.9–24.9%])	69 (24.2% [19.2–29.2%])	0.211
Limb	31 (15.8% [10.7–20.9%])	65 (22.8% [17.9–27.7%])	0.059
Polytrauma	30 (15.3% [11–19.6%])	48 (16.8% [12.4–21.3%])	0.653
**Injury severity (AIS; median and IQR)**			
Head and face	4 (IQR 3–5)	4 (IQR 2–5)	0.234
Chest	3 (IQR 2–4)	3 (IQR 3–4)	0.826
Abdomen and pelvis	2 (IQR 1.75–3)	2 (IQR 2–2)	0.892
Spine	2 (IQR 2–2.25)	2 (IQR 2–3)	0.231
Limbs	2 (IQR 2–2)	2 (IQR 2–2)	0.757
**Injury Severity Score**	16 (IQR 8–25)	13 (IQR 8–24.5)	0.533

AIS: Abbreviated Injury Score; CI: confidence interval; IQR: interquartile range.

## Discussion

This study suggests that advancing age is a predictor of injury pattern and severity after a fall down a flight of stairs. There appears to be two distinct groups of patients who present after falling down a flight of stairs: a younger population in whom the fall is commonly due to alcohol and an older group in whom alcohol is a less likely association. Significantly, the injury pattern in older patients is different and they are more likely to suffer polytrauma. The risk of falling down a flight of stairs may be increased in this older group as a result of frailty and mobility problems, but this paper was unable to explore this particular assumption. While previous studies have documented injury patterns in patients who have fallen down a flight of stairs, none specifically compared older and younger patients.^5,10^

The high incidence of head, chest and spine injuries in our study concurs with that seen previously in patients who fall downstairs.^2,3^ However, no studies compared injury patterns between different age groups with this injury mechanism. Similarly, while the higher incidence of injuries, polytrauma and ISS in older patients has been reported in older major trauma patients, this is the first time it has been reported after this particular mechanism of injury.^11–13^ These findings have implications for the approach to patients with this injury mechanism. Trauma teams and emergency physicians should suspect multiple and serious injuries in older patients who present after falling down a flight of stairs, even if they do not show overt signs on presentation. This is particularly important with spinal and head trauma, both of which can present late and with minimal signs in older people.^14,15^

These results may have some bearing on the use of imaging in patients who present after falling down a flight of stairs: we suggest that clinicians should have a low threshold for using whole body computerised tomography (WBCT) in older patients with this type of injury. While the indications for WBCT are not universally agreed, one of the main purposes of using this investigation is the detection of multiple injuries in trauma patients.^16,17^ Our study suggests that polytrauma is significantly more likely in older patients after a fall down a flight of stairs. These differences in injury pattern should guide the investigation of patients who have fallen down a flight of stairs. Previous research also suggests that older patients with serious injuries are less likely to present with abnormal vital signs, such as a low GCS or low systolic blood pressure.^14,18^ In light of this, the routine use of WBCT in patients aged 65 and older who have fallen down a flight of stairs may be justified. However, more research is needed before such a recommendation is widely implemented.

The association between alcohol intoxication and head injuries in patients who have fallen down a flight of stairs has been documented elsewhere^5,10^ and this study also found an increase in head and neck injuries, including traumatic brain injury (TBI), in younger intoxicated patients. In this group of patients, it may therefore be prudent to have a high index of suspicion for TBI and a low threshold for CT of the brain. Unlike the differences seen between older and younger patients who have fallen down a flight of stairs, there was no significant difference in ISS or the presence of polytrauma when intoxicated and sober patients were compared. Therefore, while the more liberal use of CT of the brain may be reasonable in these patients, our data do not support the routine use of WBCT in all intoxicated patients who have fallen down a flight of stairs. However, a high index of suspicion of injury and a low threshold for the use of non-invasive radiological investigations is warranted in all intoxicated trauma patients.

## Limitations

This study is the first of its kind to specifically investigate the impact of age and alcohol intoxication on injury pattern and severity in patients who present after falling down a flight of stairs but there are limitations which should be noted. The only patients included in the database were those for whom a trauma team was activated and it is likely that some patients who fell down stairs may not have activated a trauma call and therefore been included; however, the practice at AUH is that falls down stairs generally trigger trauma team activation, as they are considered a ‘fall from a height’. ‘Intoxication’ was defined as a history or clinical suspicion of alcohol intoxication rather than a blood alcohol level. While this was only considered once other medical causes for altered consciousness had been excluded, there is still the chance that a purely clinical definition may have led to some inaccuracy in diagnosis. However, other authors have demonstrated a good correlation between the clinical assessment of intoxication and blood alcohol levels.^19^

## Conclusion

This study suggests that patients who present as major trauma standbys after falling down a flight of stairs are a heterogeneous group. They include older, generally sober patients, who are more likely to sustain multiple injuries, and younger patients, who are more likely to be intoxicated, and are at a higher risk of head injuries. While this study was conducted on major trauma patients, we believe that the findings are likely to be generalisable to all patients who present to hospital after falling down a flight of stairs. Age and alcohol intoxication should be taken into account when assessing these patients, as they both impact on injury pattern and guide the use of investigations in these patients. A multicentre trial is needed to confirm the findings in a larger population.

## Compliance with ethical standards

Ethical approval was gained from the HRA via the IRAS for use of the Aintree University Hospital Trauma Database in anonymised research in March 2014 (IRAS project ID 140559). All patient data were collected anonymously, and no patient identifiable information was kept within the research database. The Research Ethics Committee therefore decided it was not necessary to obtain individual patient consent from each patient in the database.
